# Malaria preventive therapy in pregnancy and its potential impact on immunity to malaria in an area of declining transmission

**DOI:** 10.1186/s12936-015-0736-x

**Published:** 2015-05-26

**Authors:** Andrew Teo, Wina Hasang, Louise M. Randall, Holger W. Unger, Peter M. Siba, Ivo Mueller, Graham V. Brown, Stephen J. Rogerson

**Affiliations:** University of Melbourne, Department of Medicine (Royal Melbourne Hospital), Melbourne, VIC Australia; Victorian Infectious Diseases Service, The Doherty Institute, Melbourne, VIC Australia; Papua New Guinea Institute of Medical Research, Goroka, Papua New Guinea; The Walter and Eliza Hall Institute of Medical Research, Melbourne, VIC Australia; Barcelona Centre for International Health Research (CRESIB), Barcelona, Spain; The Nossal Institute for Global Health, The University of Melbourne, Melbourne, VIC Australia

**Keywords:** Malaria, Antibody, Papua New Guinea, Pregnancy, Immunity, Azithromycin, Sulfadoxine-pyrimethamine, Chloroquine, IPTp, ITN

## Abstract

**Background:**

Regular anti-malarial therapy in pregnancy, a pillar of malaria control, may affect malaria immunity, with therapeutic implications in regions of reducing transmission.

**Methods:**

Plasma antibodies to leading vaccine candidate merozoite antigens and opsonizing antibodies to endothelial-binding and placental-binding infected erythrocytes were quantified in pregnant Melanesian women receiving sulfadoxine-pyrimethamine (SP) with chloroquine taken once, or three courses of SP with azithromycin.

**Results:**

Malaria prevalence was low. Between enrolment and delivery, antibodies to recombinant antigens declined in both groups (*p* < 0.0001). In contrast, median levels of opsonizing antibodies did not change, although levels for some individuals changed significantly. In multivariate analysis, the malaria prevention regimen did not influence antibody levels.

**Conclusion:**

Different preventive anti-malarial chemotherapy regimens used during pregnancy had limited impact on malarial-immunity in a low-transmission region of Papua New Guinea.

**Trial registrations:**

NCT01136850

## Background

Pregnant women are susceptible to *Plasmodium falciparum* infection, and malaria in pregnancy (MiP) increases the risks for mother and baby [1]. Antibody to placental-binding infected erythrocytes (IEs) can protect against MiP and its consequences [2, 3]. Intermittent preventive therapy during pregnancy (IPTp) and insecticide-treated bed nets (ITN) can reduce the impact of MiP [4], but could impair development of pregnancy-associated immunity [5]. Current data on the impact of malaria prevention on the acquisition of pregnancy-associated malarial antibodies is largely restricted to African settings. It was hypothesized that multiple courses of IPTp during pregnancy may be associated with impaired development of pregnancy-associated malaria immunity, and this was tested in a cohort of pregnant women from Madang, Papua New Guinea (PNG).

## Methods

### Ethics approval

Ethics approval was obtained from the PNG Institute of Medical Research’s Institutional Review Board (08.15), the PNG Medical Research Advisory Council (05.03, 10.50) and the Human Research Ethics Committee of Melbourne Health (2001.016, 2008.162). Women provided written informed consent.

### Study participants

Pregnant women were recruited for a malaria prevention trial in Madang, PNG, and plasma samples from a subset of these women were used in this study [6]. Participants recruited at first antenatal visit (ANC) were randomly assigned to receive one course of sulfadoxine-pyrimethamine (SP) with chloroquine (CQ) (*N* = 304), control arm, or up to three courses of SP and azithromycin (AZ) (*N* = 277), intervention arm, and followed to delivery. Women were given an ITN, if available, and usage was recorded. Paired plasma samples collected at enrolment and delivery were assayed for *P. falciparum* antibodies.

### Malariometric indices

Presence of *P. falciparum* infection (enrolment, delivery) was determined by light microscopy (LMS) and quantitative polymerase chain reaction (qPCR) of peripheral blood films, from placental impression (LMS, qPCR), and by examination of placental histology. Placental malaria was classified as acute, chronic or past infection [7].

### Parasite and cell cultures

The laboratory-adapted *P. falciparum* lines CS2 (placental-binding) and E8B-ICAM (endothelial-binding), and THP-1 monocyte-like cells, were cultured as described [8].

### Assays of IgG to schizont extract, merozoite antigens and measles haemagglutinin

Samples were assayed for immunoglobulin G (IgG) antibodies to recombinant *P. falciparum* antigens and measles haemagglutinin protein by enzyme-linked immunosorbent assay (ELISA) as described [8]. In brief, microtitre plates were coated with schizont extract from CS2 (1/2000), MSP2 from FC27 (0.5 μg/ml), MSP3 from 3D7 full ectodomain (2 μg/ml), PfRH2 from 3D7 (0.5 μg/ml) and measles haemagglutinin (1 μg/ml; Abcam, Melbourne, VIC, Australia). Test plasma (1/1000, in duplicate) was added, followed by incubation with peroxidase-conjugated goat anti-human IgG (1/2500; Merck Millipore, Kilsyth, VIC, Australia). The reaction was developed and optical density was determined at 405 nm.

### Phagocytosis of infected erythrocytes

The level of opsonizing IgG antibody was determined as before [8]. In brief, 30 μL of purified trophozoite-stage IEs were stained with ethidium bromide, and opsonized with 3.3 μl of plasma for 1 h, followed by incubation with THP-1 cells for 40 min. Phagocytosis was stopped and unphagocytosed IEs were lysed, followed by fixing the THP-1 cells in 2 % (*w/v*) paraformaldehyde. The cells were acquired using a HyperCyt® CyAn flow cytometer (Beckman Coulter). Data are represented as percentage of THP-1 cells containing ingested IEs. For all assays, plasma from six adults with no history of malaria exposure was included. Discordant samples were re-run, using published rules [9].

### Antibody responses during pregnancy

To assess changes during pregnancy, the mean antibody levels from paired samples collected at enrolment and delivery were compared, and the same rules were applied as described [8]. In brief, samples were categorized based on their adjusted mean variance: 1) <20 % with mean difference of <10 %, no change; 2) >20 % with mean difference of >10 %, either decrease or increase in antibody responses.

### Statistical analysis

The main interest of analysis is whether preventive therapies during pregnancy had an impact on development of pregnancy related anti-malarial antibodies. Data were analysed with Stata version 13.0 (StataCorp) and GraphPad Prism v5 (GraphPad Software, Inc). Differences in population mean ranks of paired continuous non-parametric variables, antibody responses, were evaluated using the Wilcoxon signed-rank test. Categorical variables were compared using the *χ*^2^ tests. Multiple linear regression analysis was performed to determine associations between continuous and categorical variables on antibody, and adjusted analyses included treatment arms, maternal characteristics, and interaction variables.

## Results

### Patient characteristics

Participant characteristics (SP-CQ *n* = 304, SP-AZ *n* = 277) were similar between groups. Most women receiving SP-AZ (82 %) had three courses. The prevalence of malaria infection was low at enrolment and delivery (Table 1).

**Table 1:** Study population characteristics of Papua New Guinean women

Characteristic	Single course of CQ and SP treatment (*n* = 304)	Three courses of SP and AZ treatment (*n* = 277)	*P*-value
Age, years	24.0 (21.0–28.0)	24.0 (21.0–28.0)	0.2
Weight at enrolment, kg	55.0 (50.0–59.0)	53.0 (49.0–59.0)	0.6
MUAC at enrolment, cm	23.0 (22.0–25.0)	23.0 (22.0–25.0)	0.5
Gravidity			0.4
Gravida 1, *n* (%)	108 (35.5)	92 (33.2)	
Gravida 2, *n* (%)	91 (29.9)	88 (31.8)	
Gravida 3, *n* (%)	105 (34.5)	97 (35.0)	
Overall Bed net use			0.4
No, *n* (%)	2 (0.7)	0 (0.0)	
Intermittent, *n* (%)	89 (29.3)	82 (29.6)	
Regular, *n* (%)	213 (70.1)	195 (70.4)	
Ethnicity			0.5
Madang/Morobe	194 (63.8)	193 (69.7)	
Sepik	57 (18.8)	41 (14.8)	
Highland	26 (8.6)	19 (6.9)	
Others	27 (8.9)	24 (8.7)	
Residence			0.9
Urban	51 (16.8)	44 (16.0)	
Peri-urban	59 (19.5)	50 (18.1)	
Rural	181 (59.7)	170 (61.8)	
Migrant	12 (4.0)	11 (4.0)	
Light microscopy (*P. falciparum)*			
Enrolment (peripheral blood)	20 (6.6)	15 (5.4)	0.5
Delivery (peripheral blood)	7 (2.3)	5 (1.8)	0.7
Delivery (placental blood)	7 (2.3)	4 (1.4)	0.2
qPCR (*P. falciparum)*			
Enrolment (peripheral blood)	31 (10.2)	24 (8.7)	0.8
Delivery (peripheral blood)	14 (4.6)	8 (2.9)	0.1
Delivery (placental blood)	7 (2.3)	1 (0.3)	0.1
Placental histology			0.1
Uninfected	171 (81.8)	170 (81.0)	
Infected^a^	38 (18.2)	40 (19.1)	

Data represented as median and interquartile range, unless otherwise indicated

*AZ* azithromycin, *CQ* chloroquine, *SP* sulfadoxine-pyrimethamine, *MUAC* mid-upper arm circumference

^a^Placental malaria was defined as histological evidence of acute, chronic, or past infection

### Antibody to recombinant antigens

**Fig. 1:**
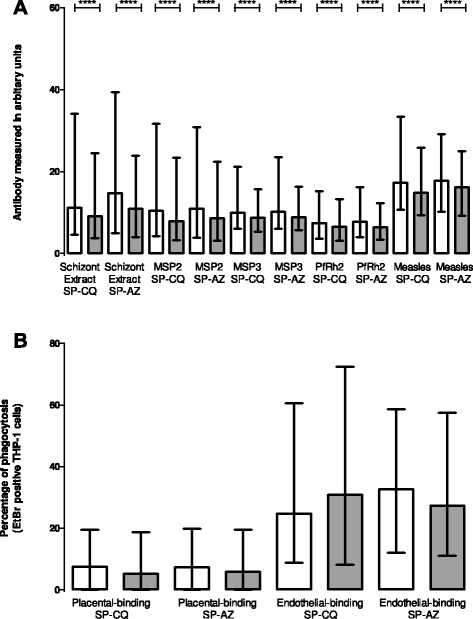
Levels of immunoglobulin G (IgG) antibody in PNG pregnant women against *Plasmodium falciparum* antigens over the course of one pregnancy. White bars- pregnant women recruited at first antenatal visit, grey bars pregnant women at delivery. Pregnant women on sulfadoxine-pyrimethamine (SP) and chloroquine (CQ) [*N* = 304], and on SP and azithromycin (AZ) [*N* = 277]. **a** Levels of IgG antibodies to schizont extract, PfRh2, MSP2, MSP3 and measles antigen presented as arbitrary units. **b** Levels of opsonising IgG antibodies to variant surface antigens of placental-binding and endothelial-binding IEs, presented as percentage of THP-1 cells that have ingested IESs (percentage phagocytosis). Wilcoxon signed-rank test, ****p < 0.0001. Columns represents IQR and error bars shows 95 % CI

Median antibody levels to schizont extract and merozoite antigens did not differ by treatment arm at delivery (schizont extract z = −0.4, PfRh2 z = −0.8, MSP2 z = 0.2, MSP3 z = −0.3, all *p* > 0.05). There was a significant decline in median level of antibodies against schizont extract, MSP2, MSP3 and PfRh2 between enrolment and delivery (all *p* < 0.0001) in both treatment arms. Antibody to measles haemagglutinin (control) showed a similar significant decline in both treatment arms (Fig. 1a).

### Opsonizing antibody to infected erythrocytes

Opsonizing antibodies to placental-binding and-endothelial-binding IEs did not vary by treatment arm at delivery (CS2 z = −0.8, E8B-ICAM z = 0.6, both *p* > 0.05). In contrast to decline in antibodies to recombinant antigens (above), median levels of opsonizing antibodies to both placental-binding and-endothelial-binding IEs did not change significantly between enrolment and delivery in either treatment arm (Fig. 1b).

### Changes in individual antibody responses by treatment arm between enrolment and delivery

When differences in individual responses were compared between enrolment and delivery, for each treatment arm, similar numbers of women experienced changes in antibodies to schizont extract, MSP2 and MSP3. Significantly more women on SP-CQ than SP-AZ experienced an increase in antibody response to PfRh2 (8.3 vs 3.6 %; *p* = 0.05), and in opsonizing antibody to endothelial-binding IEs (29.6 vs 19.1 %; *p* = 0.004). Interestingly, significantly more women on SP-AZ than SP-CQ experienced an increase in opsonizing antibody to placental-binding IEs (17.8 vs 12.0 %; *p* = 0.03; Table 2).

**Table 2:** Changes in antibody responses against *P. falciparum* antigens in the Papua New Guinean cohort during the course of pregnancy

Variable (*n*)	SP + CQ control arm (*n* = 304)	SP + AZ intervention arm (*n* = 277)	*P* value
IgG to schizont extract			0.7
Decrease, *n* (%)	70 (23.2)	67 (24.3)	
No change, *n* (%)	198 (65.6)	184 (66.8)	
Increase, *n* (%)	34 (11.3)	25 (9.1)	
IgG to MSP2			0.6
Decrease, *n* (%)	63 (20.9)	49 (17.8)	
No change, *n* (%)	217 (71.9)	203 (73.6)	
Increase, *n* (%)	22 (7.3)	24 (8.7)	
IgG to MSP3			0.8
Decrease, *n* (%)	55 (18.2)	53 (19.1)	
No change, *n* (%)	220 (72.8)	203 (73.3)	
Increase, *n* (%)	27 (8.9)	21 (7.6)	
IgG to PfRh2			**0.05**
Decrease, *n* (%)	**34 (11.2)**	**39 (14.1)**	
No change, *n* (%)	**244 (80.5)**	**228 (82.3)**	
Increase, *n* (%)	**25 (8.3)**	**10 (3.6)**	
Opsonizing IgG to E8B-ICAM			**0.004**
Decrease, *n* (%)	**72 (23.9)**	**60 (21.7)**	
No change, *n* (%)	**140 (46.5)**	**164 (59.2)**	
Increase, *n* (%)	**89 (29.6)**	**53 (19.1)**	
Opsonizing IgG to CS2			**0.03**
Decrease, *n* (%)	**39 (13.0)**	**47 (17.0)**	
No change, *n* (%)	**226 (75.1)**	**180 (65.2)**	
Increase, *n* (%)	**36 (12.0)**	**49 (17.8)**	

Data represented as numbers and percentage, *P*-values are also shown

*AZ* azithromycin, *CQ* chloroquine, *SP* sulfadoxine-pyrimethamine

Significant associations (*p* < 0.05) highlighted in bold

### Effect of treatment arm on antibodies at delivery: adjusted analysis

After adjusting for confounding and interaction variables, there were no significant differences in antibody levels at delivery between treatment arms (Table 3). Antibody levels at delivery were associated with rural residence (for schizont extract, MSP2 and MSP3), and with highlander heritage (schizont extract and MSP2), all *p* < 0.05. There was also a gravidity-dependent increase in opsonizing antibody to both endothelial-binding and-placental-binding IEs, both *p* < 0.01. Importantly, there was no evidence of the intervention arm modifying levels of malarial antibodies in higher gravidity women and women from different areas and ethnicity.

**Table 3:** Relative antibody responses against *P. falciparum* antigens at delivery by treatment arm in Madang, PNG, adjusted for confounding and interaction variables

Variables	IgG schizont extract		IgG PfRh2		IgG MSP2		IgG MSP3		Opsonizing IgG E8B-ICAM		Opsonizing IgG CS2	
	Coeff (95 % Cl)	*p*	Coeff (95 % Cl)	*p*	Coeff (95 % Cl)	*p*	Coeff (95 % Cl)	*p*	Coeff (95 % Cl)	*p*	Coeff (95 % Cl)	*p*
Intervention arm^a^	−3.0 (−13.4, 7.4)	0.6	−3.6 (−10.9, 3.7)	0.3	−7.4 (−17.9, 3.1)	0.2	2.6 (−7.0, 12.2)	0.6	1.0 (−14.2, 16.2)		−7.4 (−19.4, 4.5)	0.2
Gravidity												
1												
2	−2.0 (−8.3, 4.3)	0.5	−4.6 (−9.1, 0.2)	0.06	1.0 (−5.44, 7.42)	0.8	−0.7 (−6.6, 5.1)	0.8	**50.3 (42.8, 57.9)**	**<0.0001**	**9.3 (2.5,16.1)**	**0.007**
3	1.6 (−4.5, 7.6)	0.6	−0.5 (−4.8, 3.8)	0.8	1.9 (−4.3, 8.1)	0.6	5.72 (−0.04, 11.4)	0.06	**32.8 (25.6, 40.1)**	**<0.0001**	**24.3 (17.9, 30.8)**	**<0.0001**
Overall bed net use												
No												
Intermittent	−1.8 (−6.0, 2.4)	0.4	−0.5 (−3.4, 2.5)	0.8	−1.6 (−5.9, 2.7)	0.5	0.3 (−5.6, 10.5)	0.9	1.2 (−3.8, 6.2)	0.6	−2.6 (−7.2, 1.9)	0.3
Regular	−13.0 (−45.2, 19.2)	0.4	−5.3 (−28.1, 17.6)	0.7	−12.0 (−45.0, 20.9)	0.5	−2.5 (−32.7, 27.6)	0.9	25.4 (−13.1, 63.9)	0.2	−10.6 (−45.0, 23.8)	0.6
Residence												
Urban												
Peri-urban	0.3 (−8.4, 8.8)	0.9	−1.3 (−7.1, 5.9)	0.7	1.5 (−7.2, 10.2)	0.7	2.0 (−5.9, 10.0)	0.6	2.2 (−10.4, 14.9)	0.4	−1.9 (−11.8, 7.9)	0.7
Rural	**8.6 (1.2, 15.9)**	**0.02**	5.0 (−0.2, 10.0)	0.06	**8.5 (1.2, 16.0)**	**0.02**	**8.5 (1.7, 15.2)**	**0.01**	−4.1 (−14.8, 6.6)	0.7	−0.4 (−8.8, 8.0)	0.9
Migrant	13.0 (−1.3, 27.3)	0.07	−0.1 (−10.1, 10.0)	0.9	13.3 (−1.1, 27.7)	0.07	−1.1 (−14.3, 12.1)	0.9	6.4 (−14.4, 27.3)	0.5	2.7 (−13.7, 19.1)	0.8
Ethnicity												
Madang/Morobe												
Sepik	−4.7 (−11.0, 11.0)	0.2	0.3 (−4.7, 5.2)	0.9	−5.4 (−12.6, 1.7)	0.1	1.7 (−4.8, 8.2)	0.6	−1.6 (−11.9, 8.7)	0.8	4.7 (−12.8, 3.4)	0.2
Highlands	**−12.3 (−21.9, −2.6)**	**0.01**	−0.5 (−7.2, 6.2)	0.9	**−10.9 (−20.6, −1.2)**	**0.03**	−1.0 (−9.9, 7.7)	0.82	−3.7 (−17.7, 10.4)	0.8	−6.4 (−17.4, 4.7)	0.4
Others	−4.4 (−13.9, 5.2)	0.4	−2.5 (−9.3, 4.2)	0.5	−7.5 (−17.0, 2.2)	0.1	1.3 (−7.5, 10.1)	0.8	−2.4 (−16.5, 11.7)	0.9	−2.6 (13.5, 8.3)	0.6
SP-AZ-gravidity^a^												
1												
2	−5.6 (−14.77, 3.56)	0.2	7.1 (0.57, −13.56)	0.1	−6.5 (−15.90, 2.87)	0.2	−3.5 (−12.10, 5.06)	0.4	−6.5 (−17.48, 4.50)	0.3	−0.3 (−10.1, 9.5)	1.0
3	−5.5 (−14.3, 5.6)	0.3	1.2 (−5.1, 7.5)	0.7	−7.6 (−16.7, 1.5)	0.1	−7.5 (−15.8, 0.8)	0.08	−5.6 (−16.2, 5.0)	0.3	0.01 (−9.5, 9.5)	1.0
SP-AZ-residence^a^												
Urban												
Peri-urban	5.0 (−7.7, 17.6)	0.4	2.1 (−6.7, 11.0)	0.6	2.9 (−9.9, 15.7)	0.7	−1.1 (−12.8, 10.6)	0.9	−8.2 (−26.8, 10.3)	0.4	8.4 (−6.1, 23.0)	0.3
Rural	2.7 (−8.2, 13.6)	0.6	2.7 (−5.0, 10.3)	0.5	7.5 (−3.5, 18.5)	0.2	−3.9 (−14.0, 6.1)	0.4	−2.8 (−18.7, 13.1)	0.7	7.7 (−4.8, 2.2)	0.2
Migrant	1.2 (−19.5, 21.9)	0.9	7.2 (−7.3, 21.7)	0.3	−5.63 (−26.55, 15.29)	0.6	3.7 (−15.43, 22.78)	0.7	−17.6 (−47.8, 12.6)	0.3	0.5 (−23.3, 24.2)	1.0
SP-AZ-ethnicity^a^												
Madang/Morobe												
Sepik	0.02 (−11.0, 11.00)	1.0	1.9 (−5.9, 9.6)	0.6	5.1 (−6.0, 16.2)	0.4	−4.6 (−14.7, 5.6)	0.4	−1.6 (−17.7, 14.4)	0.8	11.5 (−1.1, 24.1)	0.07
Highlands	−1.0 (−16.0, 14.0)	0.9	−1.3 (−11.8, 9.2)	0.8	1.6 (−13.6, 16.7)	0.8	−5.53 (−19.4, 8.3)	0.4	−2.8 (−24.7, 19.1)	0.8	6.7 (−10.6, 23.9)	0.5
Others	0.5 (−14.2, 13.1)	0.9	3.3 (−6.3, 12.9)	0.5	6.6 (−7.2, 20.5)	0.4	−3.6 (−16.2, 9.0)	0.6	−1.0 (−21.1, 19.2)	0.9	−3.6 (−19.3, 12.1)	0.7

Data represented as coefficients and 95 % confidence interval (Multiple linear regression models), *P*-values are also shown

*SP* sulfadoxine-pyrimethamine, *AZ* azithromycin, against control group (SP-CQ), *CQ* chloroquine

^a^Refers to relative antibody responses in intervention group (SP-AZ). A positive coefficient implies an increase of antibody levels. A negative coefficient implies a decrease of antibody levels. Significant associations (*p* < 0.05) highlighted in bold

## Discussion

Malaria prevention during pregnancy reduces exposure and may affect the acquisition of malaria immunity [4, 5]. In these PNG women, levels of antibody to merozoite and schizont antigens declined during pregnancy whether women received single dose of SP-CQ or multiple doses of SP-AZ.

The reduction in median levels of antibody to schizont extract and several recombinant merozoite antigens between enrolment and delivery may be due to reduced exposure and/or effective clearance and protection by SP-AZ or SP-CQ and ITN. Other possible contributions include the low prevalence of infection and maternal haemodilution, as indicated by a concomitant decline in antibody to measles haemagglutinin. In contrast, there were no significant differences in median levels of opsonizing antibodies to placental-binding IEs or to endothelial-binding IEs. These observations are consistent with a previous study from PNG demonstrating maintenance of opsonizing antibodies during lower force of infection [8]. Antibodies to merozoite antigens may have a shorter half-life than antibodies to IEs [10]. This could explain differences observed, if repeated exposure is required to maintain anti-merozoite antibodies.

There are many different assays for measuring potentially protective anti-malarial antibody. In Ghana, antibodies to recombinant placental-binding *P. falciparum* erythrocyte membrane protein 1 (PfEMP1) proteins increased during pregnancy and declined after delivery, suggesting that these antibody responses are transient in the absence of infection [11]. The lack of rise in opsonizing antibodies to placental-binding IEs may reflect the lower force of infection in this study, but lack of decline in these circumstances suggests that opsonizing antibodies might be more stable than IgG to recombinant antigens [8]. In Ghana, the levels of antibodies to recombinant endothelial-binding PfEMP1 proteins did not vary during pregnancy [11], similar to the present findings for opsonizing antibodies to endothelial-binding IEs.

The relative stability of functional opsonizing antibodies even with low force of infection, compared with IgG directed to recombinant antigens, makes their levels a potential measure of longer lasting immunity to MiP. Whether functional properties of antibodies to IEs, including opsonizing activity and inhibition of placental binding [2] are the best correlates of protective immunity requires further exploration. Longitudinal studies of functional antibodies to merozoites [12] during pregnancy would also be of great interest.

Anti-malarial prevention strategies may affect the development of pregnancy-associated malaria immunity [5, 13], but in the present study the change in opsonizing antibody to placental-binding IEs did not differ between treatment arms, after controlling for confounders and explanatory variables. The disparity between the current study and others may be explained by the differences in endemicity, high ITN coverage in this cohort, and differences in assays used. Malaria antibodies vary during pregnancy [9, 11], and it was observed that analysis at group level concealed heterogeneity in responses among individuals. For some antigens, the variations in individual women’s antibody responses between enrolment and delivery differed by treatment arm. A greater proportion of women on SP-CQ experienced increases in antibody to PfRh2 and in opsonizing antibody against endothelial-binding IEs than women on SP-AZ, consistent with increased exposure associated with the less effective intervention. Supporting this, in the parent study SP-AZ decreased parasite prevalence at delivery by microscopy and placental histology [14]. Although multiple courses of anti-malarials during pregnancy may decrease malaria exposure, the impact on immunity in the context of low prevalence of infection and high ITN use appears modest.

In contrast to antibody to endothelial-binding IEs, levels of opsonizing antibodies to placental-binding IEs were more likely to increase or decrease (rather than remaining constant) in the SP-AZ arm compared with the SP-CQ arm, consistent with previous observations [8]. This reflects the complex dynamics of pregnancy-specific malarial immunity during a single pregnancy [9–11]. Assaying at multiple time points during pregnancy may assist better evaluation of pregnancy-associated immune status.

In the adjusted analyses, factors influencing malarial antibody levels at delivery did not vary between the treatment arms. Increasing gravidity is associated with higher opsonizing antibodies to IEs consistent with gravidity-dependent acquisition of pregnancy-specific immunity [2]. Rural women had higher antibodies to schizont extract, MSP2 and MSP3 than women from urban areas, whereas highlands-born women tended to have lower antibody levels to schizont extract and MSP2. These differences, which are indicative of more malaria exposure with increasing gravidity and in women residing in the rural areas, and lower exposure in the highlands-born women, were independent of treatment arm. Anti-malarials could have a greater impact on malaria immunity in settings of lower ITN usage and higher prevalence of infection.

The current study may have underestimated potential benefits of prevention. First, the low malaria prevalence may have reduced the power to observe any potential impact of different treatment arms on the acquisition of pregnancy-associated malaria immunity. Second, although high ITN coverage may reduce the need for intensive IPTp and, consequently, reduce selection pressure on the parasites [15, 16], high ITN coverage may have further reduced malaria exposure in this study, obscuring a possible effect of drug regimen on immune responses. Finally, the time for which women participated varied between three and six months; earlier implementation of malaria prevention might have had a greater impact on immunity development. Studies from higher-transmission areas, spanning most or all of gestation, are more likely to demonstrate the impact of malaria prevention on the acquisition of pregnancy-associated malaria immunity.

## Conclusions

In conclusion, IPTp with multi-doses of SP-AZ or a single-dose SP-CQ had similar impacts on pregnancy-associated malaria immunity, possibly due to the low prevalence of infection and extensive ITN coverage observed. The decline in merozoite and schizont antibodies observed between enrolment and delivery supports this. This study also demonstrated an apparent maintenance of functional opsonizing antibodies, thought to be important for prevention of morbidity, which deserves further evaluation in other regions and in the context of other preventive measures to elucidate the optimum policy in preventing MiP in such regions.

## Abbreviations

SP: Sulfadoxine-pyrimethamine
MIP: Malaria in pregnancy
IPTp: Intermittent preventive therapy during pregnancy
ITN: Insecticide-treated bed nets
PNG: Papua New Guinea
ANC: First antenatal visit
CQ: Chloroquine
AZ: Azithromycin
LMS: Light microscopy
qPCR: Quantitative polymerase chain reaction
IgG: Immunoglobulin G
ELISA: Enzyme-lined immunosorbent assay
Ies: Infected erythrocytes
PfEMP1: *P. falciparum* erythrocyte membrane protein 1
